# Non-rigid illusory contours and global shape transformations defined by spatiotemporal boundary formation

**DOI:** 10.3389/fnhum.2014.00978

**Published:** 2014-12-16

**Authors:** Gennady Erlikhman, Yang Z. Xing, Philip J. Kellman

**Affiliations:** Department of Psychology, University of California, Los AngelesLos Angeles, CA, USA

**Keywords:** illusory contours, spatiotemporal interpolation, spatiotemporal boundary formation, perceptual organization

## Abstract

Spatiotemporal boundary formation (SBF) is the perception of form, global motion, and continuous boundaries from relations of discrete changes in local texture elements (Shipley and Kellman, 1994). In two experiments, small, circular elements underwent small displacements whenever an edge of an invisible (virtual) object passed over them. Unlike previous studies that examined only rigidly translating objects, we tested virtual objects whose properties changed continuously. Experiment 1 tested rigid objects that changed in orientation, scale, and velocity. Experiment 2 tested objects that transformed non-rigidly taking on a series of shapes. Robust SBF occurred for all of the rigid transformations tested, as well as for non-rigid virtual objects, producing the perception of continuously bounded, smoothly deforming shapes. These novel illusions involve perhaps the most extreme cases of visual perception of continuous boundaries and shape from minimal information. They show that SBF encompasses a wider range of illusory phenomena than previously understood, and they present substantial challenges for existing models of SBF.

## Introduction

How do we perceive the boundaries of objects? This is, first of all, a question of what information is available in the optical input to the eyes. Often, objects differ from their backgrounds or from other objects in surface characteristics; these differences produce discontinuities in luminance, color, or texture in their retinal projections. In ordinary environments (as opposed to pictures), there also tend to be depth discontinuities at object boundaries. These are manifest optically in stereoscopic disparities at boundaries as well as through changes in relative motion of points along a boundary during object or observer motion.

In many situations, however, discontinuities in these stimulus properties are insufficient to reveal the complete boundaries of objects. Most pervasive are cases of occlusion, in which parts of an object's boundaries do not project to the eye due to a nearer, interposed object. Likewise, under conditions of camouflage, object surface properties may closely match properties of the background. Perception of complete objects in such cases depends on interpolation processes, as have been investigated in the perception of partially occluded and illusory contours and objects (e.g., Michotte et al., 1964; Kanizsa, 1974; Grossberg and Mingolla, 1985; Kellman and Shipley, 1991 for a review, see Shipley and Kellman, 2001). Experiments and models in this area have revealed a great deal about how the visual system goes beyond local visual information and uses spatial and temporal relations among physically specified edges to determine the occurrence and positions of interpolated edges.

These processes are used pervasively to overcome complex patterns of occlusion in ordinary environments; yet perceiving object boundaries can be even more difficult. Suppose that *no* oriented edge fragments are visible. This can occur in camouflage, or more frequently, under dark viewing conditions, where a few sparse elements or features of a surface may be all that can be detected. Gibson et al. (1969) showed that even under such impoverished circumstances, objects can be perceived to have continuous boundaries. Under conditions of relative motion of objects and observers, an object and its background undergo accretion and deletion of texture elements. Accretion and deletion of even sparse texture elements on a farther surface by a nearer one can give rise to the perception of continuous boundaries, shape, and the relative depth of the two surfaces (Gibson et al., 1969; Kaplan, 1969; Braunstein et al., 1982; Rogers and Graham, 1983; Prazdny, 1986; Yonas et al., 1987; Andersen and Cortese, 1989; Ono et al., 1989).

**Figure 1:**
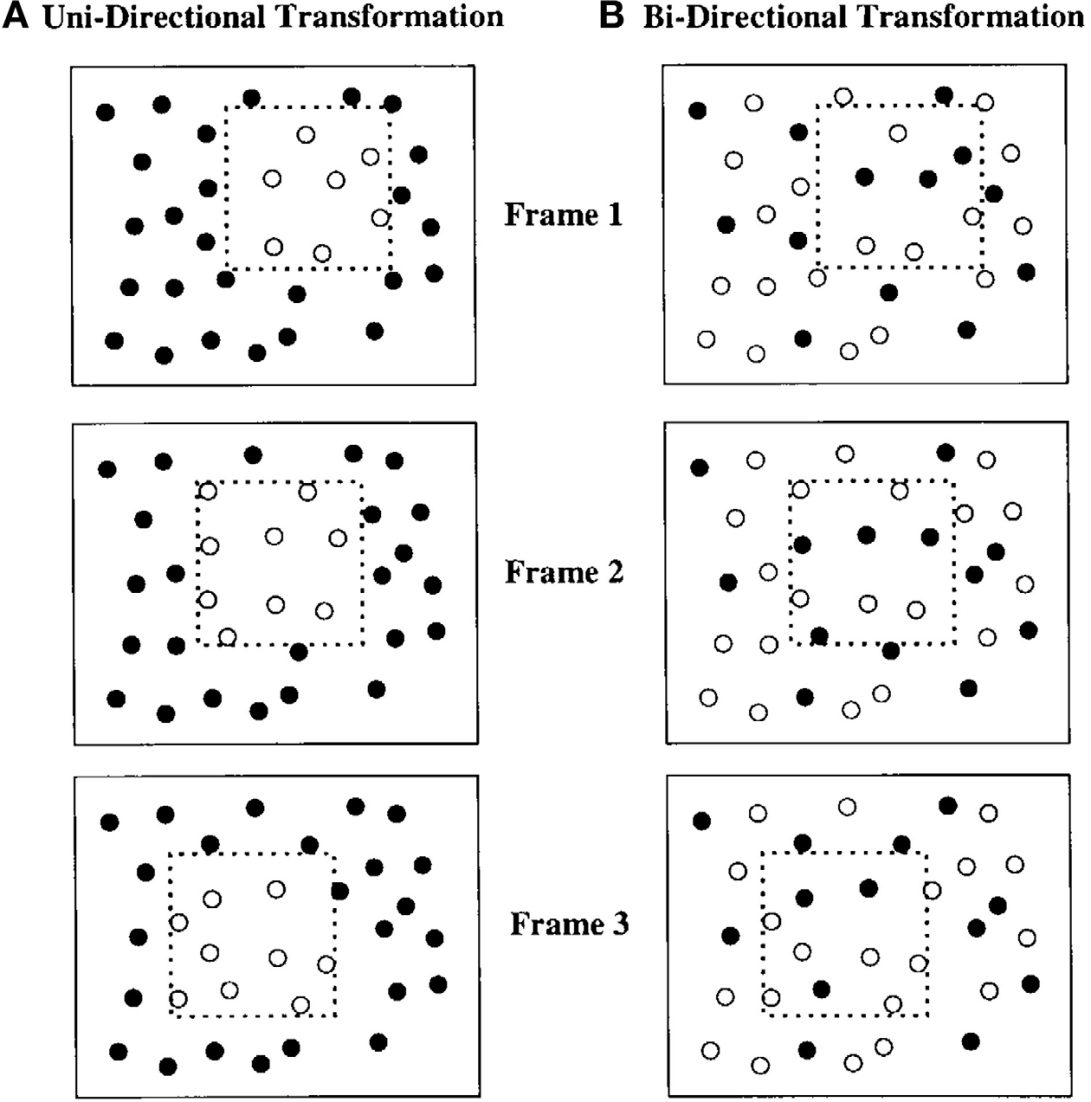
**Depiction of a square “pseudosurface” or virtual object moving over a field of circular black elements**. **(A)** All elements inside the square region are in one state and all those outside are in another. Each individual frame contains a collection of white circles in an amorphous group. As the square moves (Frames 2 and 3), elements entering and exiting the region change states. The resulting percept is of a moving, colored region with crisply defined illusory contours. **(B)** Elements inside and outside the figure can be in either of two states. As the square region moves, elements entering or exiting the region change states. Figure from Shipley and Kellman (1993).

Shipley and Kellman (1993, 1994, 1997) revisited accretion and deletion of texture and showed that it is just one example of transformations that can serve as the input to a more general process, which they called *spatiotemporal boundary formation* (SBF). They hypothesized that the crucial information for boundaries and shape in accretion and deletion is not the gradual covering or uncovering of texture elements, but the fact that those events are encoded as abrupt transformations (*spatiotemporal discontinuities*). If this more general idea is correct, then transformations of other element properties should also be capable of producing the perception of continuous contours, shape, and relative motion. Their experiments revealed that discrete appearance and disappearance of texture elements, not just gradual covering or uncovering, produced SBF. Color change also produces SBF. Moreover, a whole range of ecologically bizarre transformations, including orientation change, position change (local element motion), and form change (of elements) also produce SBF. Figure 1 shows an example of SBF displays. All transformations are unitary and discrete, meaning that they occur instantaneously with no partial covering of the texture elements.

SBF occurs for both *unidirectional* and *bidirectional* transformations. In unidirectional displays, elements entering a specified virtual region all change their feature values in the same way. For example, in a unidirectional color change display, a virtual region moves among an array of white dots against a black background. Dots change from white to blue upon entering the virtual region and change back from blue to white upon exiting. Unidirectional color change displays have been extensively studied by Cicerone, Hoffman and colleagues, with an emphasis on perceived color spreading within such displays (Cicerone et al., 1995; Cicerone and Hoffman, 1997; Miyahara and Cicerone, 1997; Fidopiastis et al., 2000; Chen and Cicerone, 2002a,b; see also, Gepshtein and Kubovy, 2000; Cicchini and Spillmann, 2013). One feature of such displays is that in static views, a region corresponding to the virtual region (albeit with unclear boundaries) can be segregated from the background. In *bidirectional* displays, all texture elements are randomly assigned one of two feature values, so no such region is visible in any static view. Elements switch values when entering or exiting the virtual region. In a bidirectional color display with blue and white dots, when the virtual region passes over dots, the white dots turn blue and the blue dots turn white. Bidirectional displays also support SBF, producing the perception of continuous contours, complete shape, and global motion, but with no color spreading. The lack of uniform color across elements within the perceived shape's boundaries appears to prevent perceptual surface formation. Instead, ring-like objects with an empty interior region are seen. Besides homogeneous color, common motion of interior elements can also produce perception of a surface (Shipley and Kellman, 1994; Cunningham et al., 1998a,b), as in classical accretion-deletion displays.

### The aperture problem in SBF

In SBF, the only information available that can be used to recover moving contours are the positions and relative timing of abrupt element transformations. This presents a seemingly impossible version of the aperture problem. In the classic version (Wallach, 1935; Adelson and Movshon, 1982; Nakayama and Silverman, 1988, 1998; Shimojo et al., 1989), when an object's boundaries are seen through many small apertures, the visual system must determine the combined velocity structure of many spatially discrete, oriented contour segments whose global velocity is ambiguous. For each edge segment, its orientation and orthogonal motion velocity is available within the aperture. In SBF, the apertures are local elements that change discretely in some property. These changes by themselves do not produce the perception of a moving edge. Moreover, edge fragments seen through apertures provide clear orientation information and constrain the directions of its movement to a 180° range. Individual element changes in SBF provide no orientation information and no usable global motion information. Depending on the transformation used to produce SBF, there may be local orientation changes (when element orientation change is used) or local motions (when element displacement is used), but these events not only provide no information about a larger form and moving contours, they provide what would appear to be contradictory information. This more extreme version of the aperture problem in SBF has been referred to as the “point-aperture problem” (Prophet et al., 2001).

One proposed solution relies on an intersection of constraints (Shipley and Kellman, 1994, 1997; Kellman et al., 2012). Successive transformations of texture elements produce velocity signals that are determined by the spatial and temporal separation between transformation events. The velocity, orientation, and global motion direction of a region boundary are constrained by these signals. Consider several one-dimensional strips of evenly spaced texture elements at different orientations. Element transformations will be slowest along the strip that is orthogonal to the boundary of a moving virtual object that passes over the strips, revealing the boundary's orientation. Given transformations along two strips, both the velocity of the boundary and its orientation can be recovered (see Shipley and Kellman, 1994, 1997 for details).

As in the intersection of constraints solution to the typical aperture problem, this model produces a coherent output only when several constraints are met. The texture element transformations are assumed to come from (1) a single, rigid entity that is (2) moving at a constant velocity. It is also assumed that the boundary can be decomposed into piecewise linear segments for which the orientation and velocity can be determined locally and independently. Such a model has been successfully implemented for bar-like shapes whose boundaries have a single orientation and velocity along their length (Kellman et al., 2012).

Previous work has considered these constraints only in the case of unchanging shapes. Strong versions of the constraints state that SBF should not occur for transforming shapes. For example, if a shape rotates, local edge orientations change continuously. If a shape scales, it also changes the local orientation of the contours. If a shape accelerates, the assumption of constant velocity is violated. The intersection of constraints solution relies on the fact that fixed edge orientations and velocities produce specific spatiotemporal patterns of texture element transformations (i.e., which elements change and at what rate). If the pattern is constantly changing between element transformation events due to changes in edge orientation and velocity the model would be unable to recover those properties. In short, existing versions of SBF models, on the simplest account of their underlying assumptions, would work for a limited class of objects. Not coincidentally, these correspond to the objects that have been used in prior studies: rigid shapes moving with unchanging orientation and constant velocity.

Here we explore whether these limitations on SBF or its models may be arbitrary. In the real world, object motions are not limited to translation at constant velocity; objects rotate, accelerate, and scale (at least retinally). When objects rotate in depth, the retinal projection of their boundaries transform non-rigidly. In structure-from-motion (SFM) displays, we can readily see these as well as other non-rigid motions, such as the deformation of elastic objects or biological motion, even in sparse dot displays (e.g., Jain and Zaidi, 2011). Does SBF work with shapes whose boundaries are changing in orientation or size? If illusory boundaries can be seen for SBF-defined objects that rotate, scale, and transform non-rigidly, this may force a reexamination of SBF models. Perception of shapes and illusory contours under these conditions would also provide the most spectacular versions of this class of visual illusion. Moving, deforming illusory contours would be seen between stationary texture elements in the absence of any local orientation and motion information.

We report two surprising visual illusions involving SBF. In Experiment 1, SBF-defined illusory figures are seen that rotate, scale, and change velocity. Even though the displays contain only sparse texture elements such that no contour or shape information is available on any given frame, robust global form and motion is seen. In Experiment 2, observers were able to see non-rigid illusory contours produced by continuously deforming SBF-defined illusory figures. The displays demonstrate a new, easy way to create non-rigid illusory contours of arbitrary complexity.

## Experiment 1

Experiment 1 used object transformations of rotation, scaling, and acceleration to test core assumptions about SBF. SBF is thought to arise from the integration of local motion signals across space and time. Shipley and Kellman (1997) suggested that pairs of discrete element changes proximate in space and time provide the input to SBF. If viewed in isolation, such pairs of element changes would produce a perception of nearest neighbor apparent motion (Ullman, 1979). In SBF two or more vectors produced by pairs of element changes that are present within a certain spatiotemporal window are integrated to produce moving, oriented contour fragments. At a higher level, perception of object shape and continuous boundaries in SBF appears to depend on spatiotemporal interpolation processes that connect these edge fragments. Spatiotemporal contour interpolation, which has been studied in other contexts, relies on the updating of position information of contour segments that have disappeared based on a representation of their orientation and velocity. This persistence and positional updating of previously seen contour fragments in a temporary visual store allows such fragments to be integrated with contour segments that appear at a later time (Palmer et al., 2006; Palmer and Kellman, 2014).

Existing models of SBF assume that local edge orientation and velocity are fixed within the integration window (Shipley and Kellman, 1994, 1997). Both the initial formation of edge fragments, and most, but not all, studies of spatiotemporal interpolation between edge fragments, have used contours with fixed orientations and velocities, and, as this would imply, rigid shapes of unchanging size and orientation. Experiment 1 tested whether SBF operates when these parameters change.

**Figure 2:**
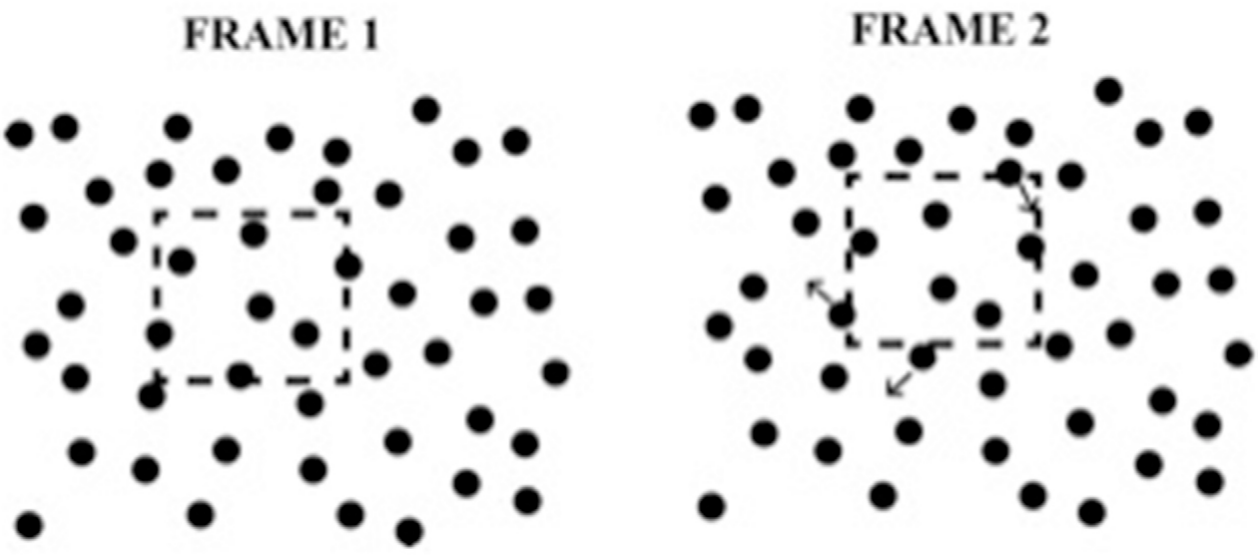
**Example of the element transformation used in Experiments 1 and 2**. The dashed region defines a “virtual object” which is not seen by the observer. As the virtual object moves, elements that enter or leave the boundary of the virtual object are displaced in a random direction.

A secondary goal of the experiment was to determine whether element transformations consisting of motions in random directions could support SBF (see Figure 2). In previous work, consistent element motions (displacement in a uniform direction of all elements upon entering the virtual object) produced SBF (Shipley and Kellman, 1993, 1994). Preliminary work in our laboratory suggested that random element motion (consistent in extent but random in direction) could also support SBF, but no prior work has used these in SBF experiments. An example is shown in Supplementary Movie 1.

A virtue of using small, random element displacements as the inducing events in SBF displays is that no static view contains any information about global shape.

As in earlier research on SBF, we used a forced-choice shape identification paradigm. The paradigm is an objective performance method, in that there was an objectively correct answer (which virtual object was used in the display) on each trial. In the absence of global shape formation, consistently accurate shape perception is not possible (Shipley and Kellman, 1993).

### Materials and methods

#### Participants

Subjects were 16 undergraduate students (3 male, mean age: 19, range: 18–21) from the University of California, Los Angeles. All participants reported having normal or corrected-to-normal vision. Subjects received course credit for participating. Experiments were approved and conducted under the guidelines of the UCLA IRB. All subjects provided informed consent to participate.

#### Apparatus

All displays were created and displayed using the MATLAB programming language and the Psychophysics Toolbox (Brainard, 1997; Pelli, 1997). Stimuli were presented on a Viewsonic G250 CRT monitor, which was powered by a MacPro 4 with a 2.66 GHz Quad-Core Intel Xeon processor and an NVidia GeForce GT120 graphics card. The monitor was set to a resolution of 1024 × 768 pixels and a refresh rate of 60 Hz.

#### Displays

Small red circles (diameter = 11.9 arcmin) were shown on a black background that filled the screen (40 × 30 cm; 25.06 × 18.92°). The total number of elements was either 200, 400, 600, or 1200. Elements were pseudo-randomly arranged by creating 100 equally sized regions and placing an equal number of elements in a random position within each region (see Shipley and Kellman, 1994). This minimized overlap between elements and ensured a nearly uniform distribution of elements across the display thereby also avoiding large, empty regions. The four element quantities corresponded to element densities of 0.42, 0.84, 1.27, and 2.53 elements per square degree of visual angle respectively. Elements covered 1.28, 2.56, 3.83, or 7.67% of the pixels in the display area.

**Figure 3:**
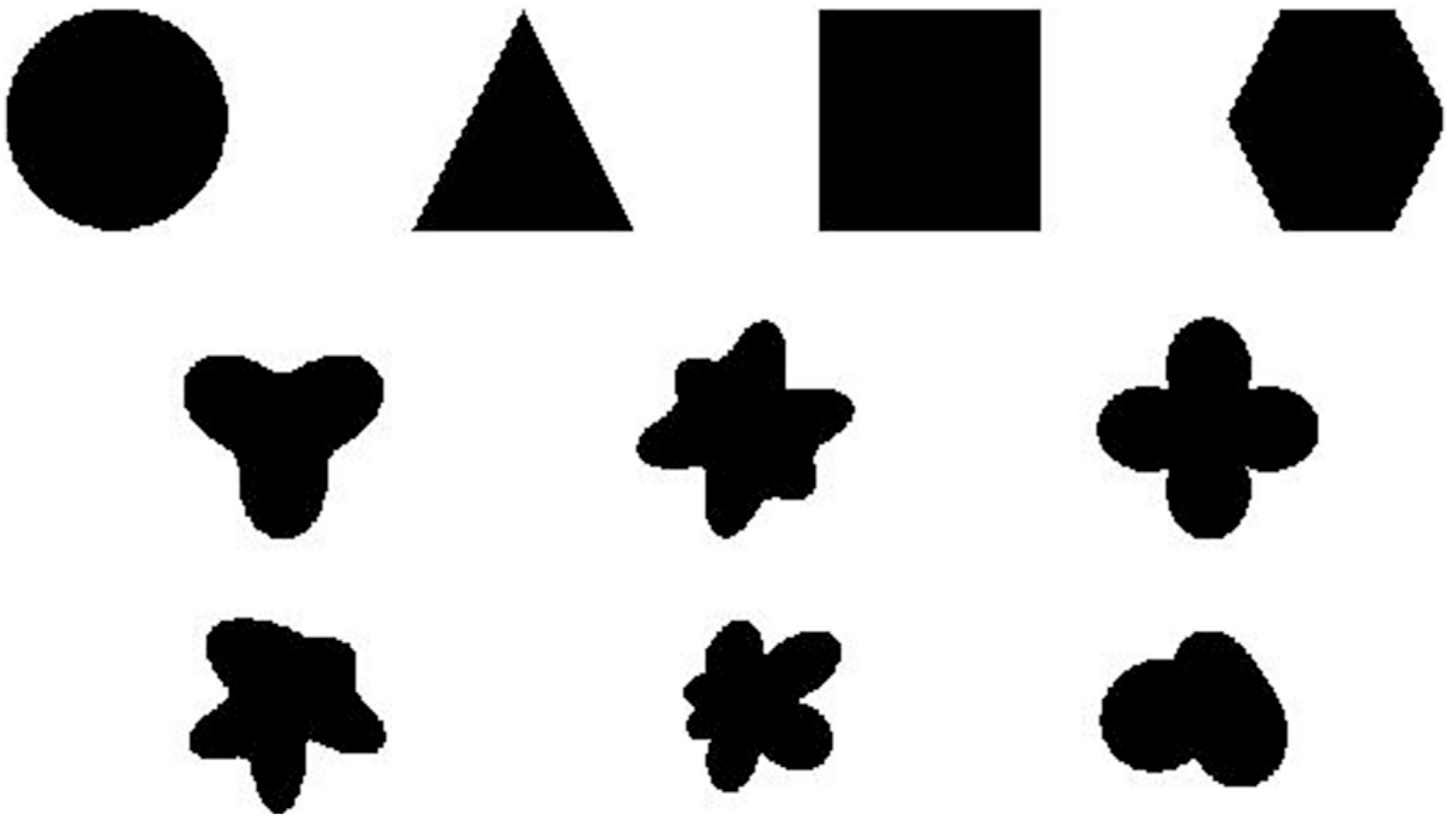
**Ten shapes used in Experiments 1 and 2**. The top four shapes are familiar, regular, and have multiple axes of symmetry. The second row contain shapes that are more unusual, but still symmetrical. The final row contains asymmetrical shapes. All shapes have approximately the same horizontal and vertical extent. They are modeled after the shapes used in Shipley and Kellman (1994). Throughout the text we refer to them as circle, triangle, square, hexagon, tri-leaf, butterfly, four-leaf, rand1, rand2, and rand3 starting from the top-left and going to the bottom-right.

We defined 10 virtual objects or “pseudosurfaces” similar to those used in Shipley and Kellman (1993)^1^. They are depicted in Figure 3. We will refer to these as virtual objects or virtual regions, while noting that they were referred to as “pseudosurfaces” in earlier work. Either label is intended to convey that the shapes are not physical entities; any static frame of the display is seen to contain only a field of undifferentiated texture elements. The shapes had varying degrees of symmetry and regularity. The virtual objects were on average 5.6 degrees of visual angle in height and width, within a range of 4.36–6.45° in either dimension. When a virtual object came into contact with an element, the element was displaced by 10 pixels (14.9 arcmin) in a random direction (see Figure 2). The displacements were large enough to be readily detectible (Shaffer and Wallach, 1966; Shipley and Kellman, 1993). When the element's original position was no longer within the boundary of the virtual objects, the element returned to that position. An element was defined as inside the virtual object if its center was on or inside of the virtual object boundary. On average, 1.74, 3.44, 5.27, and 10.51 elements transformed from frame-to-frame for each of the four element quantities respectively. An example of a scaling and rotating shape is shown in Supplementary Movie 2.

Virtual objects traveled on a circular path centered on the middle of the screen, with a radius of 4.97°. The path was divided into 360 equidistant positions which the virtual object visited them sequentially. The virtual object traveled at a rate of four positions every frame (0.35° per frame) and each frame was shown for 32.2 ms. A trial was completed when the virtual object made one complete circuit of the path. The starting position along the path was randomized across trials. A trial lasted 3 s.

As virtual objects traveled along the path, they underwent one of four possible transformations: scaling, rotation, rotation and scaling, or acceleration. In the scaling and rotation and scaling conditions, virtual objects increased or decreased in size at a rate of 1% per frame. The maximum size of a virtual object was 9.92° and the minimum size was 2.49° in any dimension. Upon reaching the size limit, scaling direction reversed. Initial scaling direction (shrinking or growing) was randomized across trials. If the virtual object was rotating, it rotated at a rate of 3° per frame in the clockwise direction. Starting orientation of the shape was always upright. In the scaling and rotation condition, both of the transformations were applied simultaneously. In the acceleration condition, on each frame, there was a 30% probability of the velocity increasing, a 30% probability of the velocity decreasing and a 40% of the velocity remaining constant. Velocity changes affected which of the 360 positions along the path the virtual object would visit. Minimum velocity was two positions per frame (compared to a base velocity of four) and maximum velocity was seven positions per frame.

#### Design

On each trial, participants performed a forced choice identification of the shape in the displays from among a fixed set of 10 alternatives. The four texture element quantities, the four shape transformation conditions, and the 10 shapes were counterbalanced in a 4 × 4 × 10 design. Each trial was repeated twice, resulting in a total of 320 trials. Trial order was randomized. Prior to the experimental trials, there were 10 practice trials. Each practice trial had the highest density of elements and no shape transformation. Each of the 10 shapes was shown once, in random order. The entire experiment lasted approximately 40 min.

#### Procedure

Subjects sat in a dark room at a distance of 90 cm from the computer monitor, with the only illumination coming from the monitor. They were given verbal and written instructions explaining that they were going to see a black screen with red dots in which an illusory shape would appear to move. Their task was to identify the shape that they had seen out of a set of 10 possible shapes. Subjects then began the practice trials. At the start of each trial, a white fixation cross appeared in the middle of a black screen for 1 s. Then, the cross disappeared and the red texture elements were shown. The virtual object began to move as soon as the elements appeared. Once the object completed a full path around the screen, a new display with an image of the 10 shapes was shown. Subjects made a response by clicking on one of the ten shapes with the mouse. A red, rectangular box appeared around the answer choice for 1.5 s to indicate the subject's response. For practice trials, feedback was provided by showing a green, rectangular, box around the correct choice. If the subject had selected the correct response, the green box surrounded the red one. In addition, the word “Correct” or “Incorrect” appeared in the top-left corner of the screen. Subjects had unlimited time to make a response. Once the practice trials were over, a message appeared on the screen, instructing subjects that the practice trials were over and that they would no longer receive any feedback.

### Results

**Figure 4:**
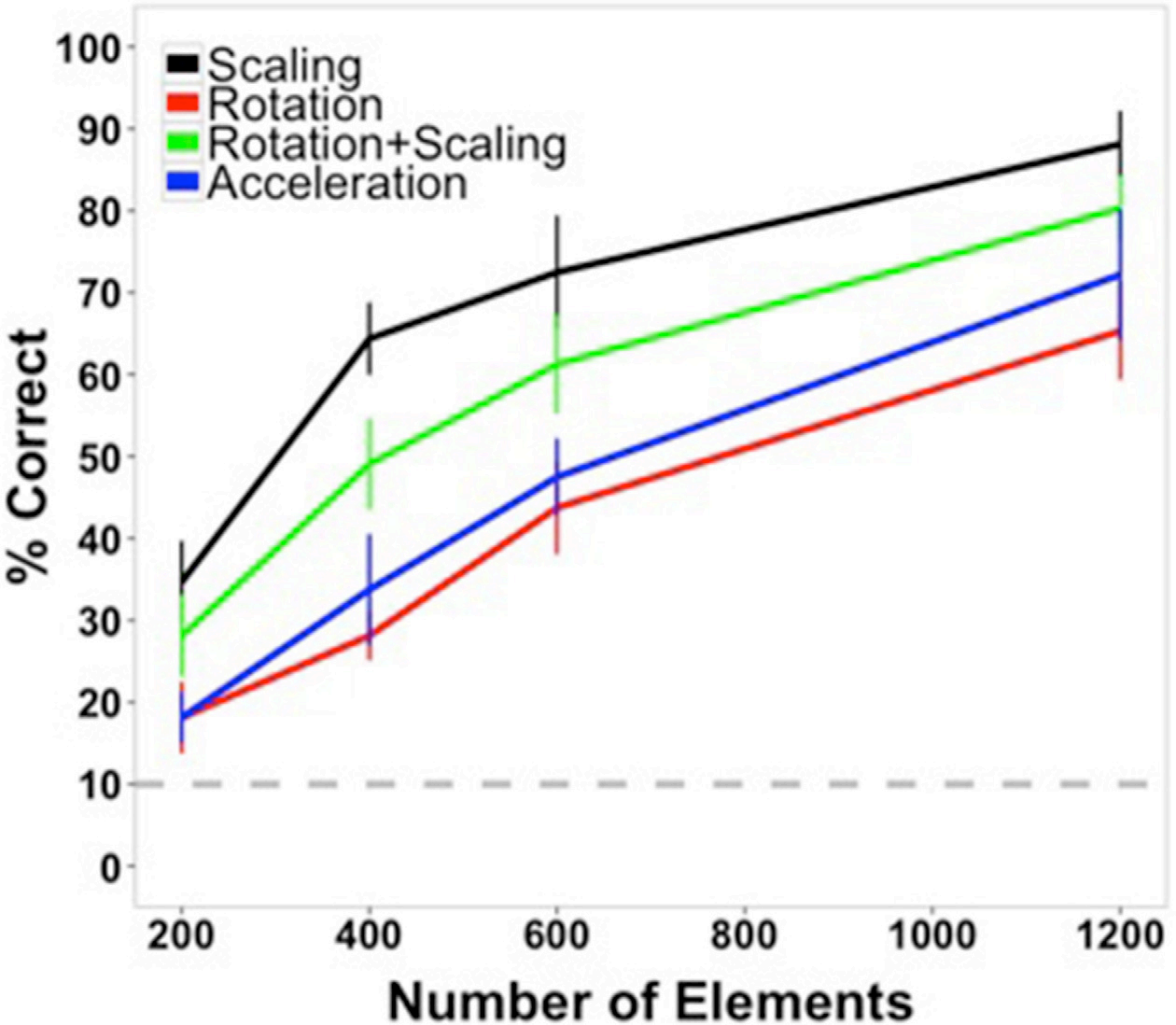
**Average accuracy data from Experiment 1 as a function of number of texture elements in the display**. Data are averaged across subjects and shapes. All transformation conditions were within-subject. Error bars indicate 95% confidence intervals. The gray, dashed line indicates chance performance (10%).

Mean accuracy data for Experiment 1 are shown in Figure 4. Highly accurate shape perception was possible under some of the conditions of the experiment, especially at the highest element density, and all conditions appeared to exceed chance accuracy. These observations were confirmed by the analyses. Accuracy data were collapsed across shapes and submitted to a 4 × 4 within-subjects ANOVA. There was a main effect of transformation type [*F*_(3, 45)_ = 90.18, *p* < 0.001, η^2^_*p*_ = 0.86], with highest accuracy for scaling shapes, followed by scaling and rotating shapes, rotating shapes, and accelerating shapes across most element quantities. There was a main effect of number of elements [*F*_(3, 45)_ = 349.36, *p* < 0.001, η^2^_*p*_ = 0.959], with accuracy improving with an increasing number of elements. There was also a significant interaction [*F*_(9, 135)_ = 2.70, *p* = 0.006, η^2^*_p_* = 0.15].

The highest accuracy was observed for the scaling condition with the largest element quantity (88.13%). Performance for this shape transformation at this number of elements was greater than all of the other transformation conditions [rotation: 65.31%, *t*_(15)_ = 6.80, *p* < 0.0001; scaling + rotation: 80.31%, *t*_(15)_ = 2.74, *p* = 0.015; acceleration: 72.19%, *t*_(15)_ = 5.08, *p* < 0.001]. For the lowest number of elements tested (200), performance in all conditions was above chance (10%) [scaling: 34.69%, *t*_(15)_ = 9.64, *p* < 0.0001; rotation: 18.12%, *t*_(15)_ = 3.64, *p* < 0.005; rotation + scaling: 28.13%, *t*_(15)_ = 7.15, *p* < 0.0001; acceleration: 18.12%, *t*_(15)_ = 5.17, *p* < 0.001].

### Discussion

The results of Experiment 1 show that boundaries and shape can be perceived from SBF displays in which the virtual objects change orientation, scale, and velocity. Moreover, these illusory figures were seen despite the transformations being displacements of individual texture elements in random directions, thereby producing incoherent local motion signals. Accurate perception of shape and the subjective appearance of continuous illusory contours bounding shapes illustrates the extreme nature of interpolation processes in SBF. Texture element transformations were spatiotemporally quite sparse in this study. There were on average 1.7, 3.4, 5.2, and 10.5 element transformations per frame, for each of the four element densities respectively. These were spread along an average shape boundary length of 17.6°. Shapes and illusory contours perceived in SBF, including the transforming shapes in this study, therefore represent perhaps the most extreme illusion involving illusory contours in terms of spatial support. Even when a number of accumulated frames are considered together, the amount of total boundary specified by local stimulus information is a very small percentage. Displays perceived in SBF represent, in an important sense, the most boundary and shape information perceived from the least stimulus input.

It is surprising that transforming virtual objects produced such robust SBF, given that changing orientation of edges in a neighborhood, changing stimulus velocity, and the changing size of virtual objects should all complicate recovery of the local edge fragments. Transforming virtual objects create two confounding problems for SBF models. First, in between successive transformations of local texture elements, the edges that caused those transformations are changing not only in their position, but also in their orientation and velocity. Since several transformation events are needed constrain the orientation and velocity of an edge, it is unclear how the visual system can relate two or more events caused by, essentially, two different edges. Second, once local edge segments are recovered, they must be interpolated in different positions along the virtual object boundary and at different times. We return to these issues in the General Discussion.

Shape identification was affected predictably by the manipulation of element quantity, improving as a function of the number of elements. Performance was best for scaling and the combination of scaling and rotation shape transformations. Accuracy may have been better in those conditions because as objects become larger, more texture element transformation events occur along the virtual object boundary. For the highest density, there were, on average, 4.64 transformations per frame when a virtual object reached its smallest size (compared to an average 10.51 transformations per frame for rotating objects that did not change size) and 18.68 transformations per frame when the virtual object was largest. However, this increase in the number of element changes scales directly with figure size, so that the number of element changes per unit of perimeter remains constant. Perhaps a more plausible account of improved performance with larger sized objects is that size may make differences between similar shapes larger and more discriminable. For example, at the highest density in the scaling condition, circles were never confused for hexagons or *vice versa*, but they were confused 17 times across all subjects at that element density when the objects were rotating without scaling.

The pattern of results in this experiment, with element position changes in random directions, was similar to experiments in which element transformations were position shifts in only one or two directions and when virtual objects were rigid and not transforming (Shipley and Kellman, 1993, 1994). In those studies, performance also increased as a function of element quantity. Since different numbers of elements were used across studies, converting the independent variable to element density per degree of visual angle allows a standard metric for comparison. We take up these comparisons after considering the results of Experiment 2 below.

## Experiment 2

Experiment 1 showed that shapes that rotate, scale, and accelerate can be accurately perceived in SBF. In Experiment 2, we further examined the types of global shape transformations that are supported by SBF. Changes in orientation, scale, and velocity are rigid transformations of the virtual object. Perhaps non-rigid transformations can also be perceived. In these displays, virtual objects smoothly morphed from one of the ten shapes used in Experiment 1 into another. Morphing continued from shape to shape until all shapes were seen. Subjects were instructed to look for a target shape (say, the triangle) in the morphing sequence and to indicate when they saw that shape (see Supplementary Movie 3).

If non-rigid illusory contours are seen in these displays, this presents a much more confounding problem for spatiotemporal interpolation. In addition to the difficulty in matching texture element transformation events with contours that are changing in position and orientation, the visual system must now deal with changes in contour curvature as the shape is morphing. Supposing that local edge segments can be somehow recovered even though the curvature of those segments changes in between transformation events, the segments must then still be interpolated. While it has been demonstrated that contour fragments that change in orientation under occlusion can be interpolated with visible ones, it is not known whether contour segments, real or illusory, can undergo changes in curvature while not visible and still be interpolated with other contour segments that are later revealed.

### Materials and methods

#### Participants

The participant group was composed of 12 University of California, Los Angeles undergraduate students (10 female, mean age = 22.75). All participants reported having either normal or corrected-to-normal vision. Participants were awarded course credit for their participation. Experiments were approved and conducted under the guidelines of the UCLA IRB. All subjects provided informed consent to participate.

#### Displays and apparatus

Since the lowest element densities in Experiment 1 made shape identification difficult when the shape was not changing, higher element quantities were used to ensure that performance was not at floor. The three element quantities used were 529, 900, and 1600. In order to accommodate the larger number of elements on the screen, texture element diameter was reduced to 7 arcmin for a viewing distance of 134.5 cm. The element quantities corresponded to densities of 2.46, 4.18, and 7.43 elements per square degree of visual angle. Elements covered 2.62, 4.47, and 7.95% of the total display area.

The same shapes were used as in the first experiment. Average virtual object diameter was 4.45°. The smallest size was 3.35° and largest was 5.03°. On average, there were 3.8, 6.47, and 11.47 element transformations per frame for each of the three element quantities respectively. As in Experiment 1, the virtual object traveled along a circular path centered on the middle of the display. The radius of the path was 3.33°. The path was divided into 120 equidistant positions. The distance between each position was 0.17°. The virtual object visited one position per frame. Each frame lasted for 33.2 ms. It took an object 4 s to make a full revolution. Starting position along the path was randomized across trials.

On each trial, the virtual object began as one of the ten shapes and smoothly morphed from one shape to another until it had become each of the 10 shapes once. Shape morphing was performed by selecting 120 equally spaced points along the contour of each shape to use as reference points. A morphing algorithm generated 99 intermediate shapes between every pairing of shapes by creating matches between the nearest contour points of the two shapes and interpolating intermediate locations. In total, there were 90 such morphing sequences, one between each pair of shapes. The first and last steps of the morphing sequence were the original, un-morphed shapes. Each intermediate morphing step therefore reflected the relative proportion of the two shapes that were being morphed. For example, on the 31st step in the morphing sequence between shapes A and B, the displayed shape was 69% shape A and 31% shape B. The entire transformation sequence from one shape to another took approximately 3.3 s.

The transformation sequences on each trial involved nine transformations between the 10 shapes. The order of shapes in the transformation sequence was randomized on each trial with the constraint that the first and last shapes could not be the target shape. Each trial lasted a maximum of 30 s. Each shape served as the target shape twice for each density, resulting in a total of 60 trials. Trial order was randomized. As in Experiment 1, there were 10 practice trials to help familiarize the participants with the task. Each of the 10 shapes was the target for one of the practice trials. The highest density backgrounds were used for all practice trials. The entire experiment lasted approximately 30 min.

#### Design and procedure

Participants were informed that the purpose of the study was to examine the perception of changing visual illusions. The stimulus was described as a morphing shape that would result from a pattern of flickering dots on the screen. At the beginning of each trial, the participant was presented with a target shape selected from one of the ten possible shapes. After a key press, the textured background appeared and the animation began. The participant was instructed to press a key when they believed the virtual object on the screen most closely resembled the target shape. The display was terminated immediately once the participant pressed the key. If no response was given during the course of the animation sequence, the trial was repeated (same target shape), but with a different shape transformation sequence. Subjects were instructed to try to make a response on the second or third viewing of a trial, and to avoid repeating a trial more often.

The first 10 trials of the experiment were practice trials at the highest density. Each of the 10 shapes was the target shape on one of the ten trials. Feedback was provided on the screen after every practice trial (“Correct” or “Incorrect”). Once the practice trials were over, the subject was informed via instructions on the screen that they would no longer receive feedback and that the number of texture elements would vary across trials.

#### Dependent measures and data analysis

A response was scored as correct if it was made while the virtual object on the screen was a morph of 50% or more of the target shape. This occurred as one shape morphed into the target shape or as the target shape began morphing into another. Since each frame corresponded to a 1% morphing of the virtual object, the range within which a response was scored as correct was 50 frames on either side of the frame that contained a 100% morph (i.e. un-morphed) of target shape.

The exact frame on which a response was recorded presumably includes time for response initiation and execution (i.e., response time). We applied a correction to account for the delay between when a relevant perceptual event caused an observer to initiate a response and when a subsequent key press was recorded. For example, a response time correction that corresponded to 30 frames would mean that if an observer initiated a response when the virtual object was a 50% morph of the target shape, then the recorded response would occur 30 frames later, when the object was an 80% morph. Likewise, a recorded response when the object was a 60% morph of the target shape would actually correspond to a response initiation 30 frames earlier, when the object was only a 30% morph.

We defined the frame that contained the 100% morph of the target shape as the *target frame*, the frame on which a key press was recorded as the *response frame*, and the frame on which the response was initiated as the *decision frame*. The response time was defined as the difference between the response frame and the decision frame. Applying a response time correction shifted the center of the window within which a response was considered correct forward in time. With no correction, the window would be centered on the target frame and would span 50 frames on either side. A 30-frame correction would shift the window forward by 30 frames so that correct responses would be those response frames that occur between 20 frames before or 80 frames after the target frame.

We considered all integer response time corrections between 0 and 50 frames. For each correction, we determined the window within which responses were correct and computed the average accuracy across all subjects and conditions. The response time correction that resulted in highest average accuracy was 12–15 frames (all times in that range produced the same accuracy). Those numbers of frames corresponded to times of 398.4–498 ms, which roughly agree with response times from object priming studies (Vorberg et al., 2003), recognition memory (Sternberg, 1969), and RSVP paradigms (Botella, 1992). The difference in average accuracy with and without the correction was less than 1% and all subsequent analyses were no different whether the correction was applied or not.

### Results

**Figure 5:**
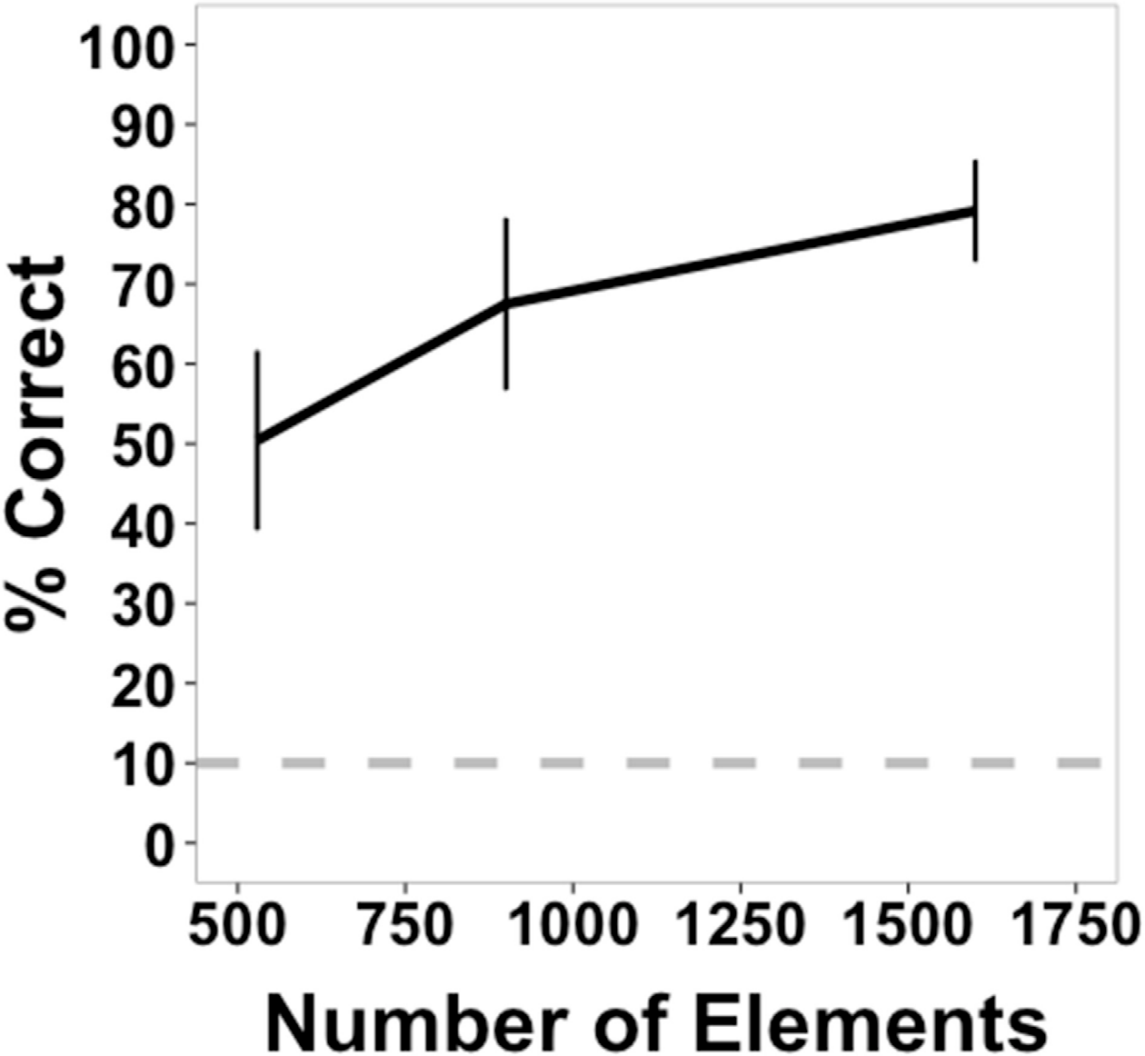
**Average accuracy data from Experiment 2 as a function of number of texture elements in the display**. Data are averaged across subjects and shapes. Error bars indicate 95% confidence intervals. The dotted gray bar indicates chance performance (10%).

Mean accuracy data for Experiment 2 are shown in Figure 5. It can be seen that successful shape identification occurred well above chance performance throughout, reaching very high accuracies at the highest element density. Accuracy data were collapsed across shapes and submitted to a One-Way, within-subjects ANOVA. There was a significant main effect of density [*F*_(2, 22)_ = 19.38, *p* < 0.001, η^2^*_p_* = 0.64]. Pairwise comparisons between the three densities revealed that accuracy at the highest density was greater than at the other two densities [high vs. medium: *t*_(11)_ =2.55, *p* = 0.027; high vs. low: *t*_(11)_ = 5.43, *p* < 0.001] and that accuracy at the medium density was greater than at lowest density [medium vs. low: *t*_(11)_ = 4.31, *p* = 0.001]. These and the results that follow were the same for analyses without the response time correction.

Accuracy data were also examined separately for each shape across the three element quantities (Figure 7). Data were collapsed across subjects since each target shape was repeated only twice per subject. Low, medium, and high in the figure legend correspond to the three element quantities (529, 900, and 1600 elements). Identification accuracy was perfect for triangles and hexagons for the largest element quantities and exceeded 90% for squares, the quad-leaf shape, and the shape Rand3 (see Figure 3 for naming conventions). Worst performance for any element quantity was for the shape Rand1 (25%). Worst performance at the largest quantity was for shape Rand2 (41.67%). Chance performance was 10%. Sensitivity (*d*') for each shape is shown in Figure 8. False alarms were counted as those trials in which a subject responded with any shape other than the target shape. As with accuracy, sensitivity was computed from data collected from all subjects. Sensitivity was highest for triangles (4.65), squares (3.82), and hexagons (4.68) for the highest density, and was relatively high for circles (3.15), quad-leaf (3.11), and Rand3 (3.35). Sensitivity decreased with decreasing element density.

A secondary analysis examined the degree to which the virtual object on the screen resembled the target shape on the decision frame (response time corrected). Recall that subjects were instructed to respond as close as possible to the target frame (the frame containing the 100% morph of the target shape). Looking only at trials in which subjects made a correct response, the number of frames between the target frame and the decision frame is a measure of the extent to which the virtual object resembled the target shape. Because there were 100 frames between the target shape and the subsequent shape in the transformation sequence, a decision on the 16th frame after the target frame would indicate that the shape on the screen was an 84% (100-16) morph of the target shape. Likewise, a decision 16 frames before the target frame would also contain an 84% morph of the virtual object. Results were not significantly different if the response time correction was not applied.

Figure 9 shows the percentage of target shape on the decision frame (response frame-15 frames) averaged across subjects as a function of element quantity. A one-way, within-subjects ANOVA found a significant main effect of density [*F*_(2, 22)_ = 5.65, *p* = 0.010, η^2^*_p_* = 0.34]. *Post-hoc*, between-density comparisons revealed that the percentage of target shape on the decision frame for the highest density (84.96% target shape) was significantly greater than the percentage for the lowest density [79.31%; *t*_(11)_ = 3.17, *p* = 0.009]. No other differences were significant. As before, these results were the same when the response time correction was not applied.

We further explored the data by distinguishing between decisions that came before the target frame and those that came after. The data are shown in Figure 10. A 2 × 3, within-subjects ANOVA found a significant main effect of decision time (before vs. after), [*F*_(1,10)_ = 10.20, *p* = 0.010, η^2^*_p_* = 0.50] and of element quantity [*F*_(2, 20)_ = 10.79, *p* = 0.001, η^2^*_p_* = 0.52]. There was also a significant interaction [*F*_(2, 20)_ = 4.52, *p* = 0.024, η^2^*_p_* = 0.31]. *Post-hoc* paired comparisons for percentage of target shape before and after the target frame revealed a difference for displays that contained the largest number of elements [*t*_(11)_ = 4.17, *p* = 0.002]. There were no significant differences for the two other element quantities.

### Discussion

The results of Experiment 2 demonstrate that illusory contours can be accurately perceived in SBF displays even when those boundaries are smoothly deforming. As in Experiment 1, this has two implications for the visual processes involved in perceiving boundaries in these displays. First, local boundary segments can change not only in orientation and velocity, but also in curvature in between the texture element transformation events that define them. Second, interpolation between boundary segments occurs even when one segment continues to deform after becoming invisible. Since events do not occur continuously along the entire boundary of the virtual object, they reveal only parts of the boundary at any given time. As transformation events reveal parts of the boundary, those newly visible regions interpolate with previously seen, but now invisible ones. This process suggests a form of representation that encodes constructed edge fragments as well as their continuing trajectories and deformations, allowing such information to be preserved and updated for combination with other fragments that appear at later times (c.f. Palmer et al., 2006; Palmer and Kellman, 2014). With the virtual object morphing from frame-to-frame, the boundary is deforming non-rigidly. If the visual system encodes an orientation, velocity, and curvature of a boundary segment at one moment as fixed values, those features may not align with a segment recovered at a later time. We return to this possibility in the General Discussion.

Comparing the two experiments, the best performance (across all shapes) in Experiment 2 (79.58%) was within the range of best performances from the four conditions in Experiment 1 (65.31–88.13%). However, element density had to be doubled to 4.18 elements per square degree of visual angle before this level of performance was achieved. One reason for this difference could be because virtual objects were smaller in Experiment 2 (average diameter of 4.45°) than in Experiment 1 (diameter 5.6°). Because element size was also smaller in Experiment 2, the total number of element transformations per frame was similar for the two largest densities in each experiment (12.85 in Experiment 1 and 11.47 in Experiment 2). Alternatively, a greater element density may have been needed in Experiment 2 to reach comparable performance because the task was harder. Responses were marked as correct only if they fell within 1.66 s of the target frame, whereas there was no response time limit in Experiment 1. In addition, some intermediate morphing stages may have appeared to be similar to other shapes. For example, morphing between a square and a circle may have resulted in intermediate morphs that resembled hexagons.

With the results of Experiment 2 in hand, we compared shape identification accuracy with the transforming and non-rigid virtual objects in Experiments 1 and 2 with shape identification accuracy in earlier work. Shipley and Kellman (1994, Experiment 3) used rigid, non-transforming shapes and local motion as the element transformation in a 10-AFC task. There were some differences from the present experiments. As mentioned earlier, we used a somewhat revised set of shapes. Moreover, we used random directions of element motion, whereas the earlier study used consistent vertical displacements. The virtual objects used in the current experiment were also larger (4.45° in diameter vs. 2°). Although comparisons are inexact, they may be informative with regard to the primary purpose of the present work: to determine whether SBF occurs robustly for transforming shapes. The data are clear in showing the SBF occurs with transforming objects, but if SBF occurs with transforming objects but is notably weaker than in non-transforming shapes at comparable element densities, it would suggest that changing orientation, shape, or velocity do impact the recovery of shape in SBF.

**Figure 6:**
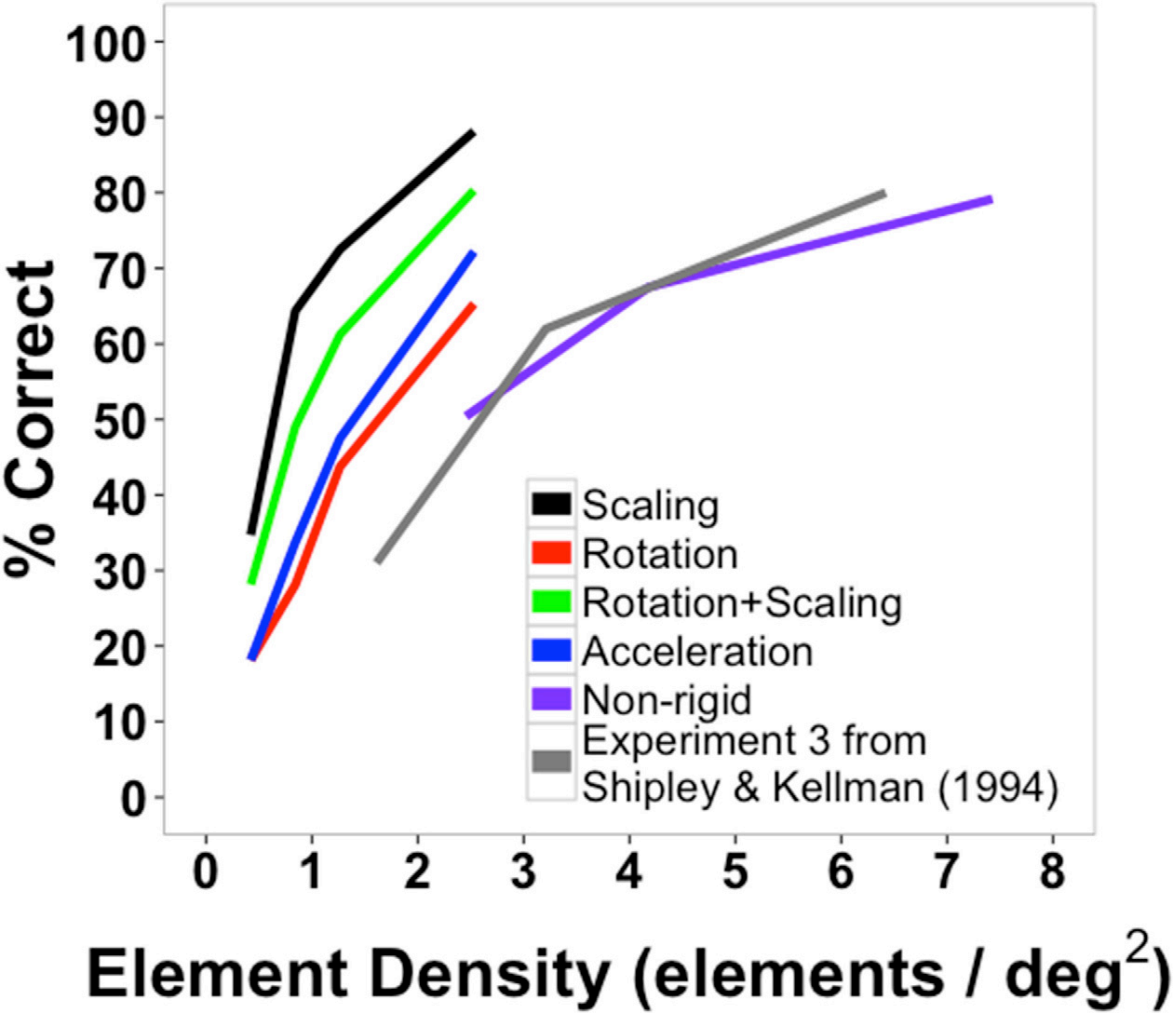
**Average shape identification accuracy from Experiments 1 (black, red, green, and blue lines) and 2 (purple line, “non-rigid”) plotted as a function of element density**. Also plotted are reproduced data from Experiment 3 from Shipley and Kellman (1994) in gray.

Figure 6 plots the data from the two current experiments along with the earlier experiment with all conditions being displayed in terms of element density (elements/deg^2^). As can be seen, performance at similar densities for deforming shapes in Experiment 2 was comparable to that of rigid, non-transforming shapes in Experiment 3 of Shipley and Kellman (1994). The four densities used in Shipley and Kellman (1994) were 1.61, 3.21, 6.42, and 12.85 elements per degree of visual angle. (Performance was not significantly different for the two largest densities and density did not exceed 6.42 in the current experiment, so accuracy for only the first three densities is shown). The densities used in the present Experiment 2 were 2.46, 4.18, and 7.43 elements per square degree of visual angle.

Figure 6 also plots the results of the transforming, rigid shapes of Experiment 1 as a function of element density. Remarkably, all of these conditions produced *better* shape identification performance than occurred with non-transforming shapes in the earlier work. The densities used in our Experiment 1 were 0.42, 0.84, 1.27, and 2.53 elements per square degree of visual angle.

For all comparable element densities, accuracy was higher in the current experiment with transforming rigid shapes than for non-transforming ones in earlier work. Even when densities were three times larger than those used in the current study, identification performance for non-transforming virtual objects was 80%, while identification accuracy for scaling virtual objects reached 88%. This difference may be because virtual objects in the current study were more than twice as large (average diameter = 5.6°) as those used previously (2.0°). That larger shapes produce better shape identification is not entirely intuitive. For displays with the same density, the number of element changes in a unit of time per unit of perimeter remains constant for a large and small display of the same shape. Also, larger shapes would tend to involve more of the retina outside of the fovea, with some attendant loss of visual acuity. It may, however, be the case that larger shapes make clearer a shape's parts and relations. We noted in the discussion of Experiment 1 that best performance observed in that study occurred in the scaling conditions, which included presentation of the largest shapes in the experiment. As suggested, the exact reason for better performance with larger shapes in SBF is not entirely clear, but one plausible hypothesis is that larger visual angles allow better definition of shape parts, resulting in improved discrimination.

**Figure 7:**
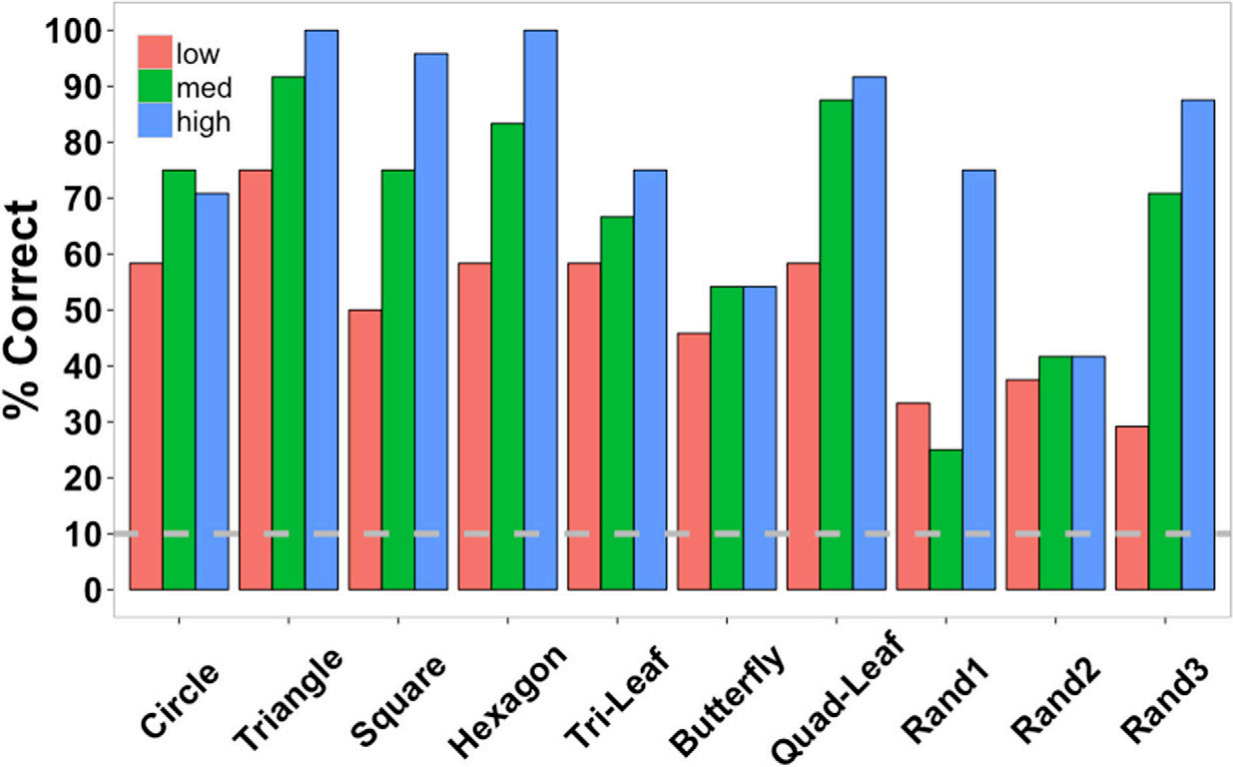
**Shape identification accuracy in Experiment 2 separated by shape and element quantity (low = 529, medium = 900, and high = 1600 elements) and collapsed across subjects**. The dashed gray line indicates chance performance. Shape names correspond to the shapes shown in Figure 3 starting at the top-left corner of the figure and proceeding left-to-right and top-to-bottom.

**Figure 8:**
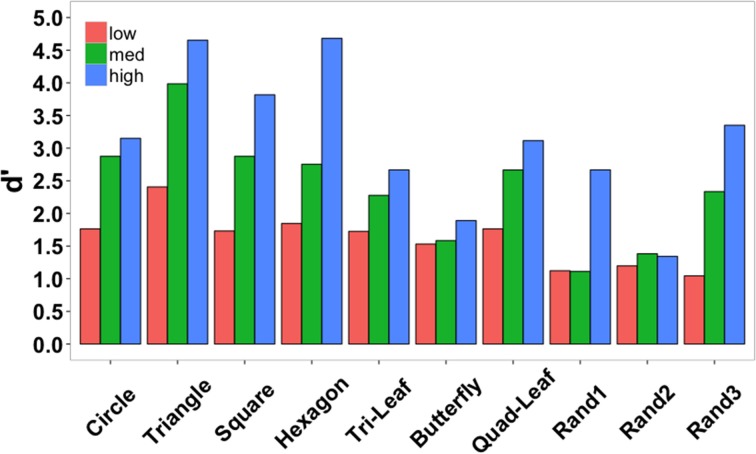
**Shape identification sensitivity (*d*') in Experiment 2 separated by shape and density (low = 529, medium = 900, and high = 1600 elements) and collapsed across subjects**.

It is clear from the data that identification accuracy varied depending on shape complexity and confusability (Figure 7). Sensitivity was greatest for triangles, squares, hexagons, quad-leafs, and shape Rand3 than for other shapes (Figure 8). Shape Rand2 appeared to be difficult to identify irrespective of the number of elements. For the lowest and intermediate element quantities, sensitivity was lowest for the shapes tri-leaf, butterfly, quad-leaf, Rand1 and Rand2. These shapes share in common regions of high curvature. Such regions require a larger number of proximal events to clearly specify the boundary. For a long, low curvature segment of a boundary, two edge fragments that are far apart would still be relatable (Kellman and Shipley, 1991). For high curvature regions, the segments would need to be from relatively nearby positions on the boundary to be relatable. Sparse texture displays would yield few recovered boundary segments.

**Figure 9:**
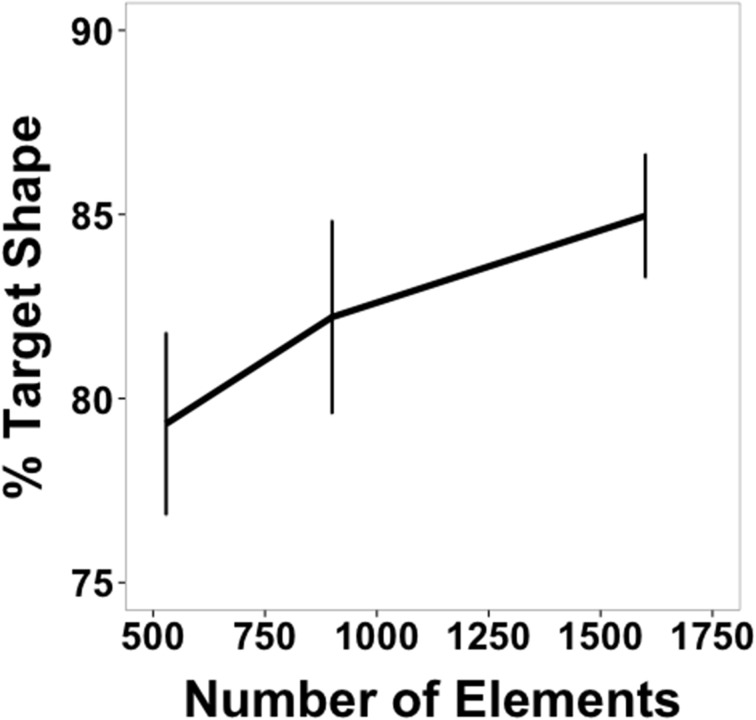
**Percentage of morph between target shape and another shape when subjects initiated a response (response time corrected, see text) as a function of element quantity**. Subjects were instructed to make a response when the figure on the screen matched as closely as possible the target shape. Values closer to 100% indicate greater response precision. Data are shown for correct trials only.

**Figure 10:**
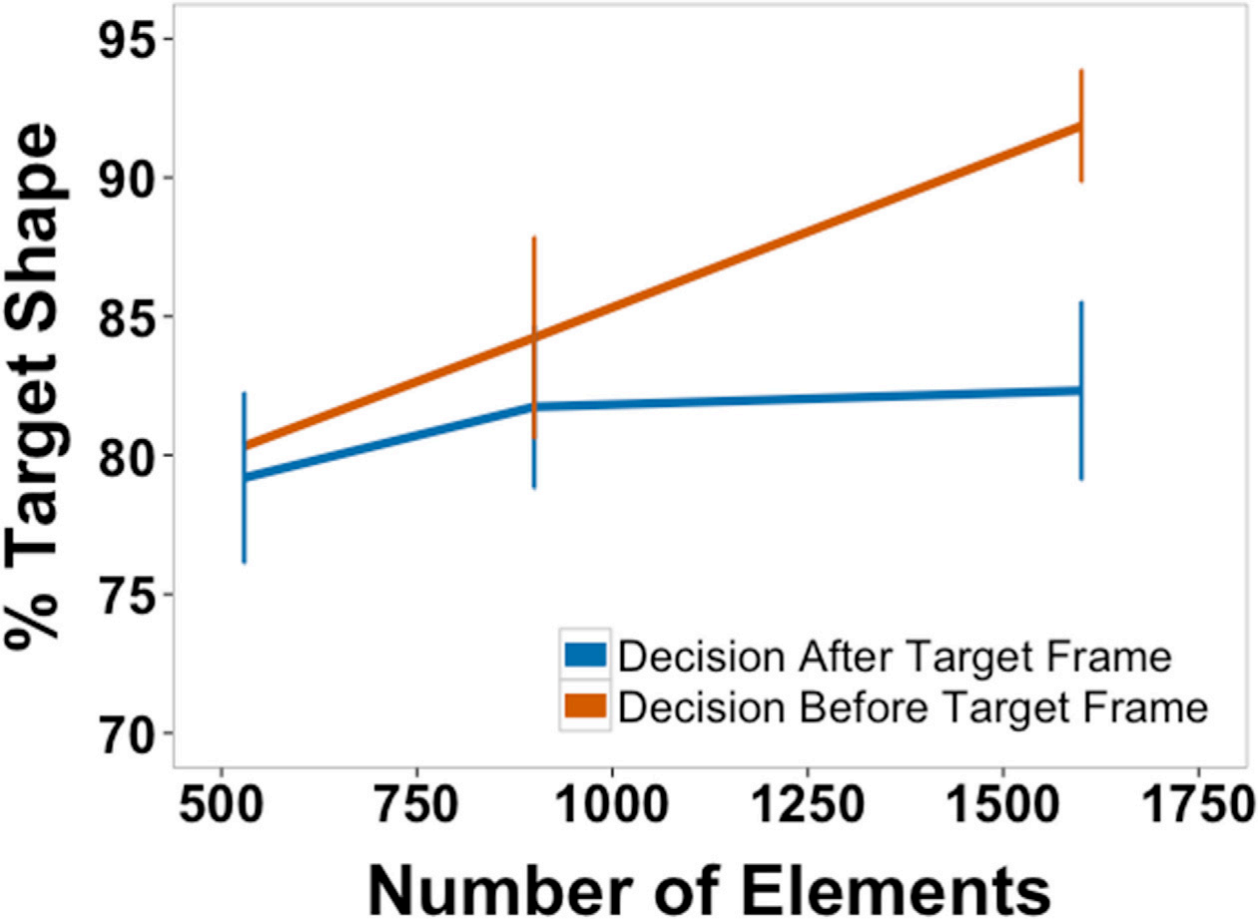
**Percentage of morph between target shape and another shape separated by whether the response came before or after the frame on which the pure target shape was presented (response time corrected, see text)**. Data are for correct trials only.

In addition to improving accuracy, element density (or quantity, as these covaried in this study) was directly proportional to response precision. Subjects tended to respond on frames closer to the target frame (the one which contained the target shape) as texture element quantity increased (Figure 9). Since the task instructions specified that subjects should respond as close as possible to the target frame, responses frames that contained shapes more closely morphed to the target shapes can be interpreted as more precise responses. Precision may have improved as a function of element quantity because subjects could more readily predict when the morphing sequence was approaching the target shape, or it could have improved because once the target frame was reached, subjects were quicker to identify the shape and respond. In order to distinguish between these two possibilities, data were split by whether responses came before or after the target frame (Figure 10). Responses after the target frame did not depend on element quantity. However, the more elements there were on the screen, the more precisely subjects could anticipate when the target frame was approaching.

## General discussion

SBF is known to produce perceived form, continuous boundaries, and global motion from discrete transformations of sparse textural elements. The use of spatially separated, sequential changes of small, sparse elements to produce these perceptual outcomes comprises an amazing spatiotemporal integration capacity of the visual system.

As with many illusions, SBF presents dual implications regarding the utility and function of perceptual processes. The events most likely to trigger SBF in real world situations are motions of objects that are poorly specified, either because of matching object and background surfaces (camouflage) or because an object is seen through multiple apertures. In these situations, SBF recovers whole objects accurately from minute bits of information spread across time. As Gibson et al. suggested in describing accretion and deletion of texture elements (the best known case of SBF), transformations produced by the relative motion of objects and surfaces carry important information deeply rooted in the optics of opaque objects, depth order, and motion. In the ways we are most likely to encounter SBF in ordinary viewing environments, SBF is a highly ecological and sophisticated mechanism for detecting what is really occurring in the environment.

But as with the processes underlying many other illusions, SBF turns out to accomplish its ecologically relevant tasks by means of mechanisms that in other cases produce ecologically impertinent outcomes. Accretion and deletion is a fact about ecological optics, but when we ask how the visual system accesses that fact, it turns out to use discrete changes in local elements—virtually *any* detectable discrete changes. This is both more and less than the original idea that the visual system detects accretion and deletion. It is much more because virtually any element transformation can provide an input into SBF, even ecologically bizarre ones such as orientation change or local displacement of an element. It is *less* than accretion and deletion because elements need not be gradually covered nor must there be any array of texture elements that move together (as will always be present during relative motion in accretion and deletion displays).

When non-ecological element transformations are used, illusory contours and shapes are perceived that cannot arise from any known physics (apart from CRT displays and clever programmers). When for example, white and blue texture elements on a black background switch values upon entering or leaving a defined, moving, virtual region, the array of changes could not be caused by any moving translucent filter nor any movement of an object seen through apertures in an occluder. The fact of a bizarre illusion, here as in other illusion contexts, lays bare the functioning of the visual processes involved. The visual processing that apprehends objects passing in front of each other from sparse information also puts together illusory shapes from abrupt changes of other kinds, such as the objects formed from local, random direction element displacements in the experiments here.

In Experiment 1, we found that the orientations, sizes, and velocities of virtual object boundaries can change between successive transformation events and still be continuously seen. In Experiment 2, SBF was also found to support changes in boundary curvature, giving rise to robust percepts of non-rigid, illusory contours. Both experiments used displays in which boundaries were perceived without accompanying filling-in or surface completion suggesting that the two processes are separable and can be studied independently. The methods described can be readily adapted to generate dynamic, non-rigid, illusory contours with arbitrary form and complexity.

### Implications for models of SBF

Perception of continuous boundaries and shape in SBF appears to depend on two processing stages. The first is to recover local edge segments from sparse texture transformation events and the second is to interpolate (connect) these segments to produce a representation of continuous contours and object shape. We consider each problem in turn.

The recovery of edges from transformation events is a difficult version of the aperture problem. Typically, local edge orientations and velocities are available in many small apertures, and the problem is to determine how they are connected and what is the global motion signal. In SBF, there is no local orientation information available since the apertures are points (the elements). The difficulty is compounded by the fact that in the displays used in these experiments, texture element transformations were element displacements in random directions, generating irrelevant and incoherent local apparent motion signals that were completely independent from the global motion of the virtual object. The relevant information for defining the virtual object boundary was solely the position and timing of transformation events. Despite these difficulties, it is possible to solve this point-aperture problem by assuming that contour segments of the virtual object boundary are rigid, moving at a constant velocity and not changing their orientation (Shipley and Kellman, 1994, 1997).

Experiments 1 and 2 demonstrated that contours can change in these properties and still support the perception of global shape and motion. Does this invalidate existing models? The answer is that models may need to be modified, but that the underlying concepts may survive. Theoretically, a local edge orientation in SBF can be recovered from three non-collinear element transformations in some local neighborhood (Shipley and Kellman, 1997). The aperture problem in SBF may get solved many times, relatively quickly, and in relatively small regions. Thus, an object may not have to be rigid or otherwise unchanging for initial edge segments to be constructed.

If the texture is sufficiently dense, the aperture problem can be solved multiple times in a small spatiotemporal window, resulting in several oriented edge representations over time. These will be small illusory contour segments. Straight or curved apparent motion might then be seen between successively recovered illusory segments that are proximal in space and time. In effect, once the aperture problem is solved for a local segment, the problem becomes a matter of detecting correspondences between sequentially recovered segments. There is no reason to suspect that this would be any different for real or illusory contours: whatever the solution to the correspondence problem that allows the matching of real contours across success frames can be applied to rotating illusory contours in SBF.

Difficulty will arise when texture displays are very sparse. In order to solve the aperture problem, multiple transformation events are needed; if the contour transforms too much between the events, then the solution might not be correct. This could explain why SBF deteriorates with decreasing element density.

Once the aperture problem is solved locally in order to recover a segment of the boundary, these boundary segments must be interpolated to produce a representation of the global shape. Since element transformation events are spatiotemporally sparse, boundary segments are recovered piecemeal, in different regions and times. This leads to the second level of processing in SBF: interpolation connecting basic contour fragments that have been formed. Because these do not appear simultaneously, the visual system needs a way of encoding recovered segments and storing and updating their representations to be interpolated with segments that are recovered at a later time. Such spatiotemporal interpolation has been found with real edge fragments in rigidly translating (Palmer et al., 2006; Palmer and Kellman, 2014) and rotating, luminance-defined edges (Kellman and Cohen, 1984), but not yet for illusory contours and not for non-rigidly deforming shapes.

According to models of spatiotemporal interpolation, when a part of an object becomes occluded, a visual, iconic representation of that surface continuous to persist for a brief time (Palmer et al., 2006). That icon is an encoding of the position, orientation, and velocity of the surface contours. If another part of the object is revealed (appears from occlusion), the visual system interpolates the relatable contours of the visual icon with those of the newly revealed object part. Interpolation is possible because the representation of the position of the now-occluded segment (the visual icon) is updated under occlusion (for a short time).

The visual system faces the same problem in SBF displays: since the aperture problem is solved locally for different areas along the virtual object boundary, edges are not recovered all at once. It is as if parts of the boundary become disoccluded whenever the problem is solved, and are occluded otherwise. The visual system must then interpolate between recovered edge segments that are visible only for short periods of time. One possibility is that the representation of occluded edges is very flexible and capable of both first- and second-order deformations. For rotating shapes, for example, when transformations along one part of the virtual object boundary reveal an edge segment, the representation of the position and orientation of that segment continues to change even when there are no further transformations to support its perception. When the aperture problem is then solved again in a nearby position, the resulting segment is interpolated with the shifted and rotated representation of the past segment if the two are relatable. A second possibility is that the representation of the segment remains fixed in terms of orientation and curvature at the moment of occlusion. A snapshot is taken, and it can only be minimally manipulated. When the next segment is recovered, the two segments must fall within the range of relatability (Kellman and Shipley, 1991) in order to be interpolated. Further studies are needed to distinguish between these two possibilities.

The present studies show that SBF encompasses a wider range of illusory phenomena than previously realized. Scaling and rotating, even accelerating rigid shapes can be recovered in SBF. Even more remarkable, deforming shapes can be perceived, and recognition of a shape is possible even when it is part of a rapidly changing series of shapes. These phenomena clearly expand the envelope beyond what previous models anticipate or explain. Although we sketched an outline of how more advanced models might encompass these perceptual illusions, the current results raise more questions than they answer, and further research will be required to achieve a detailed understanding of these amazing phenomena in which the visual system does so much with so little.

## Conflict of interest statement

The authors declare that the research was conducted in the absence of any commercial or financial relationships that could be construed as a potential conflict of interest.

## Footnotes

^1^In the original stimulus set, two of the “random” shapes (corresponding to the bottom row in Figure 3) were rotated or mirror reflections of one shape. Because shapes rotated in some conditions in this experiment, we generated new shapes that were complex and shared several features with other shapes, but were not confusable when rotated.

## Supplementary material

Supplementary Movie 1 **An SBF-defined hexagon moving along a circular path with no global shape transformation**. Texture element transformations are position changes in random directions.

Supplementary Movie 2 **A scaling and rotating SBF-defined square**. Texture element transformations are position changes in random directions.

Supplementary Movie 3 **A sample of the stimulus sequence from Experiment 2**. The virtual object transforms smoothly between some of the shapes in Figure 3. Texture element transformations are position changes in random directions. A complete transformation sequence would show transformations between each of the shapes in Figure 3.
